# Inhibitory Control, Task/Rule Switching, and Cognitive Planning in Vascular Dementia: Are There Any Differences From Vascular Aging?

**DOI:** 10.3389/fnagi.2018.00330

**Published:** 2018-10-17

**Authors:** Krystallia Pantsiou, Ourania Sfakianaki, Vasileios Papaliagkas, Dimitra Savvoulidou, Vassiliki Costa, Georgia Papantoniou, Despina Moraitou

**Affiliations:** ^1^Lab of Psychology, Department of Experimental and Cognitive Psychology, School of Psychology, Aristotle University of Thessaloniki, Thessaloniki, Greece; ^2^Laboratory of Clinical Neurophysiology, Aristotle University of Thessaloniki, Thessaloniki, Greece; ^3^1st Neurology Department, AHEPA Hospital, Aristotle University of Thessaloniki, Thessaloniki, Greece; ^4^Department of Early Childhood Education, School of Education, University of Ioannina, Ioannina, Greece

**Keywords:** cold executive functions, D-KEFS subtests, early stage vascular dementia, periventricular white matter hyperintensities, vascular hypothesis of cognitive aging

## Abstract

Recent studies have shown that patients diagnosed with Vascular Dementia (VaD) exhibit deficits in executive functions. According to “vascular hypothesis of cognitive aging,” community-dwelling older adults having risk factors for vascular disease development (RVD) may suffer from cognitive decline of the same type. The aim of the study was to assess the level of specific executive functions (EF) that have been revealed as most affected by vascular abnormalities, in older adults with incipient VaD and RVD. Subsequently specific ways of EF measuring could be suggested for more accurate diagnosis of early stage VaD. The study compared three adult groups (*N* = 60): (a) patients diagnosed with incipient VaD, according to DSM-5 criteria (*n* = 20); (b) community-dwelling older adults presenting cardiovascular risk factors (RVD; *n* = 20); (c) healthy young adult controls (*n* = 20). Three types of executive functions were examined: inhibitory control, cognitive flexibility as rule/task switching, and planning. The following D-KEFS subtests were administered for their evaluation: The ‘Color-Word Interference Test,’ the ‘Verbal Fluency Test,’ and the ‘Tower Test.’ Mixed-measures ANOVA, MANOVA, and one-way ANOVA as well as Scheffe *post hoc* test were applied to the data of the scores in each condition of each test. The results showed that VaD patients had significantly lower performance in test conditions requiring switching and planning, compared to RVD group and young controls. The specific deficits of VaD patients, compared to older adults presenting RVD according to multiple-group path analyses were: more uncorrected errors in inhibition, the use of semantic knowledge primarily instead of switching ability to switch between semantic categories, as well as a lower level of movement precision in planning.

## Introduction

Recent epidemiological studies showed that Vascular Dementia (VaD) is the second most common type of dementia in Western countries after Alzheimer’s disease (AD). Specifically, it affects approximately 5.6% of people older than 60 years old (O’Brien and Thomas, 2015). A meta-analysis of the prevalence of VaD showed that 26% of dementia cases met the NINDS-AIREN criteria for VaD, with a rate for people over 65 years between 0.6 and 2.1% (Kalaria et al., 2008; Rodriguez et al., 2008; Rizzi et al., 2014; Elahi and Miller, 2017). Many studies suggest that the rates of VaD increase with advancing age, a finding which is related to both genders (O’Brien and Thomas, 2015).

The term ‘VaD’ is used to describe a group of dementias associated with vascular brain lesions, which can cause ischemic lesions including large vessel disease (e.g., CVAs and large-vessel atherosclerosis) or small vessel disease (e.g., lacunar -very small- infarcts in the deeper white matter of the brain). The use of the term is also linked to the description of dementias associated with extensive intracerebral hemorrhages or to more focal ischemic strokes (cerebral infarctions) impairing the efficient blood supply in cortical and/or subcortical brain regions, as well as to abnormalities in hemodynamic status (Reilly et al., 2010; Korczyn et al., 2012). The presence of vascular lesions in the brain has not always been associated with cognitive decline. There are cases where large ischemic lesions are not associated with cognitive deficits whereas small vascular lesions are likely to lead to cognitive decline and loss of functionality (O’Brien and Thomas, 2015). Vascular lesions have also been observed during ‘normal aging’ but they do not always cause dementia phenomena.

Hence, many researchers are concerned about the suitability of the term ‘VaD’ for the description of the aforementioned group of dementias and have suggested to replace it with the term ‘Vascular Cognitive Impairment (VCI),’ which refers to cognitive deficits induced by various cerebrovascular lesions (Hachinski and Bowler, 1993; Román et al., 2004; Erkinjuntti, 2007; Dichgans and Leys, 2017). The main reason for the suggestion of this replacement is that the ‘traditional’ criteria for defining VaD were based on, and affected by the criteria established for AD. Hence, memory loss and impairment of everyday functioning were considered diagnostic criteria for VaD whilst none of them was necessarily the case in early VaD (Bowler, 2007). Therefore, VaD could only be identified in late stages. This underestimation of VaD incidence led to the development of the new concept of VCI, the criteria of which can be applied to diagnose VaD in early stage, and subsequently to lead to timely treatment (Bowler, 2007). Similarly, DSM-5 (American Psychiatric Association, 2013) uses the term ‘Vascular Neurocognitive Disorder (VNCD).’

The VNCD is distinguished into probable or possible. Probable VNCD is diagnosed if one of the following is present: (a) clinical criteria supported by neuroimaging evidence which confirms the cerebrovascular disease; (b) cognitive deficits that are temporally related to one or more documented cerebrovascular events; and (c) an evidence of both clinical and genetic cerebrovascular disease. Diagnosis of possible VNCD occurs when although clinical criteria are met there is no available evidence from neuroimaging and no establishment of the temporal relationship of neurocognitive disorder with one or more cerebrovascular events.

### Executive Functions in Vascular Dementia

Cognitive profiles of dementias, and especially of VaD, have been best understood due to the findings of recent neuropsychological research (Lindeboom and Weinstein, 2004; Traykov et al., 2005; Jacova et al., 2007; Nelson, 2007; Epelbaum et al., 2011; Hoffman, 2013). In this context, dysexecutive impairments have been revealed as an early feature of VaD in its various types. ‘Executive Functions (EF)’ (or otherwise ‘Cognitive control’) is an umbrella term for higher-order cognitive processes, which are supported by frontal regions and their distributed networks. They are considered as higher-order abilities because they coordinate other, cognitive, affective, and motor abilities during the execution of complex tasks (Lippa and Davis, 2010; Moreira et al., 2017). At a theoretical level, they have been divided into cold or cool EF and hot EF (Lippa and Davis, 2010; Moreira et al., 2017). Cold or cool EF include ‘pure cognitive’ or logical, goal-directed abilities such as planning, inhibition, flexibility/switching, and working memory updating. Hot EF abilities are activated when affect (e.g., motivation, emotion-regulation, metacognitive feelings etc.) is involved. Nevertheless, this distinction might not be straightforward and it has been suggested that the two types of EF are rather interdependent (Lippa and Davis, 2010; Moreira et al., 2017). Cognitive psychology and clinical neuropsychology approach EF -mainly but not only in terms of measurement- in different ways. The latter has tried to develop all-inclusive tasks/tests/scales to measure EF, in terms of the various EF abilities that should be recruited to perform in a measure. On the other hand, cognitive psychology ‘supports’ the development of ‘clear’ measures of EF. In other words, it stipulates that each task should be designed to measure a primary EF as well as component functions that contribute to performance on this task (Lippa and Davis, 2010; Moreira et al., 2017).

With regards to VaD, cold EF abilities have been extensively studied, as compared to hot EF abilities, and they are revealed, along with processing speed, to be among the first cognitive abilities that decline. Regarding memory functioning, some studies showed that in VaD patients a decline is noticed in the ability to organize the information that is going to be recalled. According to them this decline is attributed to the relationship between this specific ability and EF. The aforementioned conclusion results from the findings of a study (Di Cesare et al., 2012) in which a group of VCI participants, who were outpatients with a positive history of chronic cerebral vascular disorder, were compared with healthy younger and older adults, by means of a serial learning task of concrete frequent words. Similar conclusions were obtained from another study (Levy and Chelune, 2007) in which, groups of participants diagnosed with different dementia types, including VaD, were compared with the use of a battery of tests which are consisted of learning tasks, a verbal memory test, tasks measuring attention and executive functions as well as instruments measuring affect and emotions. In a recent longitudinal study, Livner et al. (2009) investigated two aspects of episodic memory, retrospective (free and cued recall task) and prospective (task developed for the purpose of the study), in people with AD and VaD as multi-infarct or strategic dementia. Based on the findings, they concluded that deficits in prospective memory in VaD are directly related to frontal – subcortical regions. Lamar et al. (2007) studied the relationship between leukoaraiosis in patients diagnosed with AD, VaD (probable/possible), and mixed AD/VaD, and alterations in working memory capacity measured via a modified Digit Span Backward paradigm. Their results suggest that high degrees of leukoaraiosis in people with working memory deficits are connected, to some extent, to their inability to inhibit over-learned and automatic procedural memories.

In the same vein, it seems that language skills remain intact during the various stages of VaD (even in the most progressive stages) in contrast with verbal fluency. In specific, studies which compared groups of participants diagnosed with different dementia types, including VaD, as well as groups of cognitively impaired, non-demented (CIND and vascular CIND) participants, showed that people with VaD had low scores in phonemic fluency, (Duff Canning et al., 2004) a finding which was mainly attributed to dysfunction of frontal lobes (but see Jones et al., 2006). In the same vein, during the first phase of a longitudinal study (Bagnoli et al., 2012) participants with preclinical AD and preclinical VaD as multi-infarct or strategic dementia, were examined in category and letter fluency with the use of a task of early and late word generation 3 years before the dementia diagnosis. It was found that the two groups had the same performance in letter fluency. However, preclinical VaD group outperformed their preclinical AD counterparts in category fluency which is considered to be more dependent on the medial-temporal lobe.

Visuospatial skills have been assessed by the Clock Drawing Test (CDT). Mild visuospatial deficits are observed in patients with VaD. Such deficits are depicted not only in construction abilities but also in executive control function (Graham et al., 2004; Lee et al., 2009). In particular, studies in which the CDT was used to assess the visuospatial skills of patients with subcortical VaD, confirmed spatial and planning deficits in these patients. A possible interpretation of this finding associates the observed deficits with frontal-subcortical circuit dysfunction (Graham et al., 2004). Lee et al. (2009) who studied the characteristics of CDT errors in different types of dementia, including subcortical VaD, concluded that errors in VaD are mainly associated with impairments of planning functions.

Hence, most studies show that there is a relationship between VaD in its various types and deficits in executive functions (Moorhouse et al., 2010; De Witte et al., 2011). Moreover, two review studies (Gunning-Dixon and Raz, 2000; Oosterman et al., 2004) on cognition in cerebral small vessel disease (CSVD; it refers to pathological processes that affect small arteries, capillaries, and small veins in the brain), which is considered the most common cause of subcortical vascular pathology and corresponding cognitive impairment, resulted in findings supporting executive dysfunction in CSVD. However, one of them (Oosterman et al., 2004) showed that there is indeed a significant relationship between white matter lesions and executive functions but only with regards to timed tests.

Moreover, a recent review study (Vasquez and Zakzanis, 2015) aiming at examining the cognitive profile of vascular CIND, showed that, when persons with vascular CIND are compared to persons with non-vascular CIND, they demonstrate impairments in executive functions and processing speed. However, their comparison with healthy controls suggests performance decrements across a broad range of cognitive domains., Phonemic fluency, Stroop interference, Wisconsin Card Sorting Test in various versions, Trail Making Test B and B-A (switching), and Clock Drawing are the tools mostly used in the aforementioned studies (Vasquez and Zakzanis, 2015) to measure executive functions in VaD and VCI.

Nevertheless, today, diagnosis of small vessel disease, at least at the clinical level, includes executive dysfunction manifested not only by impaired capacity to use complex information and formulate plans, but also by incapability to exercise self-control (Wallin et al., 2018). In this context, new batteries, such as the ‘Executive Interview 25,’ the ‘Frontal Assessment Battery,’ the ‘INECO Frontal Screening,’ and the ‘Frontier Executive Screen’ have been developed to measure executive functions (Moreira et al., 2017). However, more research is needed, mainly at the psychometrics level in order their usefulness to be established comparatively to traditional tasks measuring the same abilities, as well as to tests of general intelligence. Moreover, future studies would be appropriate to show the usefulness of the aforementioned tools for each neurodegenerative condition (Moreira et al., 2017; Wallin et al., 2018).

Hence, the latest diagnostic criteria for VaD (American Psychiatric Association, 2013) include the presence of cognitive impairments in frontal - executive functions (Rockwood, 2002; Desmond, 2004). Deficits in executive functions are mainly associated with subcortical regions (e.g., thalamus, caudate) and their connection with frontal–subcortical circuits (Desmond, 2004; Royall et al., 2004; Moorhouse et al., 2010; Meguro et al., 2013). Greater deficits in these functions are more frequently observed in people with severe white matter lesions (Gunning-Dixon and Raz, 2003; Buckner, 2004; Nakamizo et al., 2013).

In this vein, the present study aimed at developing a small and reliable tool to differentiate diagnosis of VaD from cognitive aging due to vascular risk factors. This tool will be comprised by a battery of tests measuring specific executive functions that have been revealed as the most vulnerable dimensions of cognition in VaD.

### Vascular Aging in Community Dwelling Older Adults

The theoretical approach of the ‘vascular hypothesis of cognitive aging’ (Spiro and Brady, 2008) posits that risk factors for the emergence of vascular disease, affect primarily cognitive functions that are supported by the frontal brain regions (van den Berg et al., 2009; Jellinger, 2013; Kling et al., 2013). A disruption to fronto-subcortical network seems to be one of the main factors that contribute to decline in executive functions. The network might be damaged by white matter lesions, microbleeds affecting connecting pathways of the network, as well as lacunes or microbleeds at subcortical structures of the network (van den Berg et al., 2009; Jellinger, 2013; Kling et al., 2013).

At the conceptual level, the ‘vascular hypothesis’ (Spiro and Brady, 2011) posits that age is only a descriptive variable of cognitive declines that occur as people age. This theoretical perspective suggests health rather than age as a candidate explanatory process. The term ‘health’ includes ‘disease’ as the opposite end of a continuum starting with ‘health.’

Older adults usually have health issues, such as elevations in various risk factors for disease, higher prevalence of disease, including subclinical situations, pure control of disease, widespread or even wrong use of medication etc. Taking all these into account, the ‘vascular hypothesis’ posits that instead of focusing on diagnostic categories, such as ‘dementia,’ and introducing new ones, such as ‘Mild Cognitive Impairment (MCI),’ ‘Subjective Cognitive Impairment (SCI)’ etc., in order to move faster on diagnosis, it might be more useful to consider cognitive aging itself as a long pathophysiological process of ‘brain at risk’ (Hachinski, 2007; Spiro and Brady, 2011). Recent findings indicating that even ‘healthy’ older adults can demonstrate a degree of vascular abnormality, suggest that there is indeed a continuum of vascular degeneration in aging, which can result in different degrees of cognitive decline, ranging from trivial to clinically relevant changes (Hachinski, 2007; Spiro and Brady, 2011).

Hypertension is one of the most important risk factors for vascular dysfunction (Moss and Jonak, 2007; Knecht et al., 2009; Sharifi et al., 2011; Areosa Sastre et al., 2017). Several studies suggest that hypertension causes pathological changes in the brain before the presence of acute events (e.g., stroke). Cognitive functions that are more affected by hypertension seem to be attention and executive functions (switching mainly) (Knecht et al., 2009; Sharifi et al., 2011; Areosa Sastre et al., 2017). In the same context, the effect of diabetes mellitus on vascular function and cognitive abilities (Yen et al., 2010; Blom et al., 2013; Hugenschmidt et al., 2013; Spauwen et al., 2013; Zhang et al., 2014) has also been demonstrated by relevant research as an additional risk factor. High levels of cholesterol, homocysteine, and ethanol (Schreurs, 2010; Ehrlich and Humpel, 2012; Niu et al., 2013; Galper et al., 2015) are included to additional physiological risk factors for cerebral vascular disorders.

Given that community dwelling older adults, without a diagnosis of dementia or MCI, usually present risk factors for developing vascular disease, it was considered useful to compare this group with patients diagnosed with incipient VaD, since the existence of risk factors could be a prodromal stage to the development of VaD.

### The Purpose and the Hypotheses of the Study

The aim of the study was to assess the level of some executive functions that have been revealed as the most affected by vascular abnormalities, (van den Berg et al., 2009; Moorhouse et al., 2010; De Witte et al., 2011; Jellinger, 2013; Kling et al., 2013) in patients with incipient VaD and community dwelling older adults presenting vascular risk factors, in order to suggest specific ways of measuring the executive functions for a more accurate diagnosis of VaD at the neuropsychological level. Since we could hardly find any healthy older adults, presenting no vascular risk factors to participate in the healthy controls’ group of the study, and as our aim was to reveal and estimate the extent of the potential declines in executive functions due to vascular risk factors and VaD, we decided to include a group of healthy young adults in the sample of the study. We considered it useful to assess young adults’ performance as an ‘index’ of the best possible performance in the tests, since it is well-established in the literature of lifespan development that fluid intelligence, including cognitive control, is at the highest possible level in this age-group (Salthouse, 2010; Spiro and Brady, 2011).

The hypotheses of the study were formulated as follows:

Hypothesis 1: we hypothesized that older adults diagnosed with incipient VaD would show a lower performance in *inhibition* tasks, compared to community dwelling older adults who are at risk of developing vascular disease. Both older adult groups were expected to have a lower performance in the same tasks compared to a healthy young adult group.
Hypothesis 2: Similarly, it was expected that patients with incipient VaD would show a lower performance in tasks requiring a *combination of inhibition and switching abilities* mainly, compared to older adults with risk factors for vascular disease. Both older adult groups were expected to have a lower performance in the same tasks compared to healthy young adult group.
Hypothesis 3: *Planning* is a complex process which integrates into the theoretical framework of cognitive control: in fact, it represents, among others, *the combined use of inhibition, switching, and updating processes.* Thus, it was expected that older adults with a diagnosis of incipient VaD would show a lower performance in planning tasks, compared to community dwelling older adults who are at risk of developing vascular disease. Both older adult groups were expected to have a lower performance in the same tasks compared to healthy young adult group.

## Materials and Methods

### Participants

The sample comprised a total of 60 adults (29 males, 31 females). Their age ranged from 20 to 85 years (*M* = 57.73, *SD* = 25.26). There were three groups: (a) healthy young adult controls (*n* = 20, 6 men and 14 women, age range: 20–25 years, *M* = 22.75, *SD* = 1.58); (b) community dwelling older adults who are at risk of developing vascular disease (RVD, *n* = 20, 13 men and 7 women, age range: 71–85 years, *M* = 75.85, *SD* = 4.47); (c) older adults with a diagnosis of incipient VaD. The diagnosis of VaD was made according to the criteria of probable VNCD (DSM-5, American Psychiatric Association, 2013; VaD, *n* = 20, 10 men and 10 women, age range: 68–83 years, *M* = 74.60, *SD* = 5.17).

The two groups of older adults did not differ significantly in age [*t*(38) = 0.81, *p* > 0.05] and gender [*t*(38) = 0.94, *p* > 0.05]. Female gender was overrepresented compared to male gender in the young adult group. The participants’ educational level varied; 23 participants (38.3%) had a low educational level (LEL: 0–9 years of education), 13 (21.7%) had a medium one (MEL: 10–12 years education), and 24 (40%) were highly educated (HEL: 13 or more years of education). The groups of older adults did not differ in educational level [χ^2^(2,40) = 0.120, *p* > 0.05]. In the VaD group, 11 participants had a LEL, 7 had a MEL, and 2 had a HEL. In the RVD group, 12 participants had a LEL, 6 had a MEL, and 2 had a HEL. All participants of the young adult group had a high educational level.

Young adults and older adults with risk factors came from the general population. History of psychosis and existence of addiction were exclusion criteria for the whole sample. Hypertension, hypercholesterolemia, and diabetes were also exclusion criteria for young adults. These criteria were selected because the authors wanted to ensure the absence of factors that could possibly provoke vascular disorders in this group. Moreover, none of the young participants was taking medication.

On the contrary, the inclusion criterion for older adults with risk factors (RVD) was a self-report of a diagnosis of hypertension, hypercholesterolemia and/or diabetes (one or more of the above), while a self-reported diagnosis of a growth-related cognitive disorder (any type of dementia or MCI) constituted an exclusion criterion for this group.

Older adults diagnosed with incipient VaD were recruited from the inpatient unit of Memory disorders and AD of the 1st Neurology Department of the Aristotle University of Thessaloniki which is located in the AHEPA Hospital. For the diagnosis of VaD (as probable VNCD), patients underwent clinical examination, laboratory tests (blood tests, biochemical tests, thyroid tests, B12, folic acid, etc.) and neuroimaging (CT/MRI). The inclusion criteria were the following: (a) clinical evidence that was consistent with a vascular etiology [(1) temporal relationship between the presence of cognitive deficits and the onset of one or more strokes, (2) evidence of early decline in cognitive abilities, such as complex attention and frontal-executive functions], (b) a cerebrovascular disease which could be indicated by medical history, physical examination, and/or neuroimaging examination, (c) cognitive deficits that could not be explained by another organic or brain disease, and (d) a degree of cognitive decline that did not prevent the daily functioning of the person.

In particular, the diagnosis of VaD was given after clinical and neuropsychological examination. Clinical examination consisted of neurological examination, biochemical and neuroimaging tests (MRI/CT/SPECT). Neuropsychological examination included the Mini Mental State Examination (MMSE), (Folstein et al., 1975; Fountoulakis et al., 2000) the Sort Cognitive Performance Test (SKT), (Lehfield and Erzigkeit, 1997) the Clinical Dementia Rating scale (CDR), (Berg, 1988) the Ishihara Tests for Color-Blindness, (Ishihara, 1992), and the Hamilton Depression Rating Scale (HDRS) (Hamilton, 1960). Based on these examinations, the neurologist was able to diagnose VaD.

The participants in this study were selected among the patients diagnosed with incipient VaD, by one of the authors (Prof. Vassiliki Costa). They were patients diagnosed with specific lesions in the brain. The majority of them had been diagnosed with multi-infarct dementia (bilateral thromboembolic events), some others with leukoaraiosis and decrease in brain volume. It is important to note that none of the participants had been diagnosed with severe hemorrhagic or ischemic stroke in the past, however, as it was found in the MRI, some of them suffered small ischemic attacks. The functional level of all participants was not affected in specific domains as clothing, dressing, feeding and use of toilet. 75% of the VaD patients reported that they had hypertension, 35% hypercholesterolemia, and 30% diabetes and for these reasons they were taking medication. The Geriatric Depression Scale -15 (GDS-15) (Sheikh and Yesavage, 1986; Fountoulakis et al., 1999) was used to examine the incidence of depressive symptoms. No scores indicative of depressive symptomatology were observed in the overall rating of the GDS-15 (score ≤ 5). In MMSE (Folstein et al., 1975; Fountoulakis et al., 2000) a wider band of scores (25–30) as compared to RVD group was observed. However, these values do not indicate the presence of cognitive impairment related to dementia (see **Table 1**).

**Table 1:** Individual – demographic - clinical information for each sample group of the study.

Sample Groups	YA^1^	RVD	VaD
	(*n* = 20)	(*n* = 20)	(*n* = 20)
Age (in years): M *(SD)*	22.75 *(1.58)*	75.85 *(4.47)*	74.60 *(5.17)*
Gender: male/female (*n*)	6/14	13/7	10/10
Educational level: low/middle/high (*n*)	0/0/20	12/6/2^∗^	11/7/2^∗^
Geriatric Depression Scale – 15: M *(SD)*	–	1.95 *(1.43)*	1.50 *(1.31)*
Mini Mental State Examination: M *(SD)*	–	28.90 *(0.71)^∗∗^*	27.00 *(1.48)^∗∗^*
Hypertension (%)	–	75% (self-reported)	75%
Hypercholesterolemia (%)	–	30% (self-reported)	35%
Diabetes (%)	–	40% (self-reported)	30%

*^1^VaD, older adults diagnosed with Vascular Dementia according to the criteria of probable Vascular Neurocognitive Disorder; RVD, community dwelling older adults having risk factors for vascular disease development; YA, young adults. ^∗^χ^2^(2,40) = 0.120, p > 0.05. ^∗∗^p < 0.05.*

In the RVD group, 75% reported having hypertension, 30% high cholesterol, and 40% reported a diagnosis of diabetes, and therefore they were taking medication. None of the participants in this group showed scores indicating depressive symptomatology in the GDS-15 (score ≤ 5). Another exclusion criterion used in this group was the performance in the MMSE, as scores under 24 are indicative of the presence of dementia symptoms. The scores of the participants ranged from 28/30 to 30/30.

The mean GDS-15 score of the RVD group (*M* = 1.95, *SD* = 1.43) did not differ [*t*(38) = 1.03, *p* > 0.05] from the mean GDS-15 score of the VaD group (*M* = 1.50, *SD* = 1.31). On the contrary, there was a significant difference [*t*(27.4) = 5.14, *p* < 0.05] in the mean scores of MMSE between RVD (*M* = 28.9, *SD* = 0.718) and VaD group (*M* = 27.0, *SD* = 1.48). The first group had a higher performance.

### Tools

Based on the literature suggesting early impairments of cold EF abilities, such as inhibition and planning, in VaD and VCI, and adopting a cognitive perspective for measuring EF, we chose to assess cold EF abilities, namely inhibition, cognitive flexibility/switching, and planning with the use of specific tests of the Delis-Kaplan Executive Function System (D-KEFS) (Delis et al., 2001). The D-KEFS includes tests that are administered to determine whether poor performance is due to specific impairment in EF, or impairment in lower-order cognitive abilities. Contrast measure scores, completion time, correct answers, and error analyses provided by the D-KEFS tests are factors which enable the assessment of an individual’s executive functioning (Delis et al., 2001; Homack et al., 2005)

#### Delis-Kaplan Executive Function System: Color – Word Interference Test, Standard Form – D-KEFS C-WIT, SF (Delis et al., 2001)

This test is based on Stroop’s experiment (1935) and measures the ability to inhibit a dominant and automatic verbal response. The participants have to inhibit an overlearned verbal response (e.g., reading the printed words) to generate a contradictory response (e.g., naming the color of the word). The test comprises four conditions: (a) naming the three basic colors (green, red and blue), (b) reading words written in black that are naming colors, (c) naming the dissonant ink colors in which the words are printed (inhibition), and (d) switching between naming the color of the words and reading the words (inhibition/switching). There is a time limit for each condition, i.e., 90 s in the first two conditions and 180 s for the following two. The examiner interrupts the test when the participant cannot complete the task within the given time.

There are two training sessions in each condition to ensure that participants have understood the task. In the first condition, the participant has to name the colors of squares (green, red, and blue). In the second condition, he/she has to read black-colored words naming the above three colors. The third condition comprises of color words written in incompatible color from the one they name. The participant has to name the color of the word instead of reading the word. Finally, in the fourth condition the participant has to (1) name the color of the word and (2) read the framed word instead of naming its color. The instructions for these tasks are displayed at the top left side of the stimulus booklet that is given to the participant. The test follows some discontinue rules as well. That is, the examiner administers the third condition only if the participant succeeds in finishing the first two ones the examiner is entitled to interrupt the administration of the test in case that participants have difficulty in the training sessions or if participants perform three consecutive uncorrected errors. Given that the contrast scores in this test have been found to display poor reliability, (Homack et al., 2005) it was decided that in this study, the examiner would record the time until the completion of the task with the restriction that there should not be an overrun of the time limit. The examiner had also to record the total of wrong answers in the form of corrected and uncorrected errors in each condition.

Plenty of clinical studies examined the validity of D-KEFS – C-WIT and showed that people with frontal lobe epilepsy, cardiovascular disorder, chronic kidney failure, AD, and Huntington’s disease have a low performance in the third and fourth condition (Kramer et al., 2002; Delis et al., 2004).

#### Delis-Kaplan Executive Function System: Verbal Fluency Test, Standard Form – D-KEFS VF, SF (Delis et al., 2001)

This test assesses crystallized intelligence and executive functions. It is comprised of three testing conditions: (a) phonemic fluency, (b) semantic (category) fluency, and (c) category switching. In the first condition, the participant has to say as many words starting with the letters F, A, and S as he/she can within 60 s. The rules of this condition are as follows: (1) Names of persons or locations are excluded of the words to be stated by the participants, (2) Numbers do not count as words, and (3) Derivatives of the words already stated by the participants are not credited with additional points. In the second condition, the participant is asked to generate as many words that belong to a designated semantic category (animals and men names) as he/she can within 60 s. Finally, in the third condition the participants are asked to generate as many fruit and furniture names as they can, switching between these two semantic categories within 60 s.

The score of phonemic fluency condition consists of the total sum of the correct answers for each letter. The ranking scale is from 0 to 1, each correct word is credited with 1 point and the wrong ones (words that do not fall within the rules or word repetitions) with 0. The score of the second condition consists of the total sum of the correct answers in each category. The ranking scale is the same as in the first condition, namely 1 point for each correct word and 0 points for irrelevant or repeated words. Lastly, the third condition score is derived from the total sum of correct words in each category (3a) and the total sum of correct switches between the semantic categories (3b). Correct answers are marked with 1 point while wrong ones with 0 points.

D-KEFS VF factor analysis revealed a three-factor solution. The first factor was labeled “conceptual flexibility,” the second one “monitoring,” and the third one “inhibition” (Latzman and Markon, 2010; Fine and Delis, 2011). The results showed that the first condition (phonemic fluency) of the D-KEFS VF had modest loadings on inhibition factor (0.35) in the 20–49 age range and a higher loading (0.61) in the 50–89 age range. The second condition (semantic fluency) had modest loadings on monitoring factor (0.39) in the 20–49 age range and higher loadings on inhibition factor (0.60) in the 50–89 age range. Lastly, the third condition (category switching) had an extremely high loading on monitoring factor (0.97) in both age ranges.

The test also assesses crystallized intelligence, the ability to use learned knowledge and experience. In other words, it is associated with the accumulated knowledge that is derived from the individual’s vocabulary depth and from the world knowledge.

#### Delis-Kaplan Executive Function System: Tower Test, Standard Form – D-KEFS – TT, SF (Delis et al., 2001)

In this test, participants have to make towers along three vertical pegs using five disks. Each participant has to solve the issue of the task using a specific minimum number of moves. The test consists of a wooden board with three vertical pegs, five wooden disks that vary in size and color (shades of blue), a timer, a recording and a stimulus booklet. The test comprises nine problems of increasing difficulty. Each problem has a specific time for completion, 30 s for the first three problems, 60 s for the fourth, 120 s for the fifth and sixth, 180 s for the seventh, and 240 s for the eighth and ninth problem. There are two rules in this test which are given both orally and in writing. The first rule is that only one disk can be moved at a time, and the second one is that no disk must be placed on top of a smaller disk. In each problem the examiner slides the disks onto the pegs, in a specific order, and shows the participant a picture of the tower he has to do (the final position of the disks). In the first problem, the participant has to use two disks the number of which is increased during the administration of the test. If the participant fails to solve the problem, the examiner shows him/her the solution. This aid only applies to the first and second problems. The examiner records the completion time of the first move (optionally), the total number of moves, the whole of rules violation (optionally), the completion time of the problem, and whether the problem was solved (yes or no). If the participant exceeds the time provided, the examiner interrupts the resolution of the problem and scores it with 0. The examiner interrupts the administration of the test in case that participants perform three consecutive failures (i.e., exceeded the given time, making an incorrect version of the tower, using fewer moves than the those origianally predefined).

The scores of D-KEFS TT used in this study are the following: (a) the total number of administered problems, (b) the total number of violations of the rules, (c) the total achievement score which is derived from the sum of the total of correct solutions (the scoring is based on the number of moves). The range of raw scores extends from 0 (zero problems solved) to 30 (all problems solved within the given time and with the minimum number of moves). In the present study, (d) the precision of movements was also measured, which results from the ratio of the total number of movements to the total number of the minimum predefined movements for each administered problem. This measurement assesses the state of planning awareness and the ability to learn heuristic strategies when solving problems.

D-KEFS TT is a tool which assesses complex executive functioning. Factor analysis (Latzman and Markon, 2010) showed positive loadings on spatial planning, learning rules, inhibition, and the ability to define and maintain cognitive sets. The validity of the tool has been studied by various studies in people with brain damage. A recent study showed that people with cardiovascular disease had achieved a lower overall score compared to control group (Jefferson et al., 2006; Fine and Delis, 2011).

### Procedure

The above tools were merged to create a battery. Two different versions of this battery, as far as the order of administration is concerned, were designed to avoid order effects. The battery tests were individually administrated and the length of the test administration was almost an hour. The examination took place in a quiet and comfortable environment.

Young adults and older adults presenting risk factors for developing vascular disease were recruited from the community by the first author (Krystallia Pantsiou) via convenience sampling. They were examined at a place of their own choice. Patients diagnosed with incipient VaD, were recruited from the inpatient unit of Memory disorders and AD, of the AHEPA hospital, and they were examined by the same author in an office of the unit.

### Ethics Statement

The authors assert that all procedures contributing to this work comply with the ethical standards of the relevant national and institutional committees on human experimentation and with the Helsinki Declaration of 1975, as revised in 2008. All participants participated voluntarily in the study. They were informed about the procedure and the aim of the study, and subsequently they provided their written consent for participation. The Ethics Committee of the School of Psychology of the Aristotle University of Thessaloniki, after reviewing the research protocol, confirmed that all ethical guidelines for research on human subjects were followed (011/16-06-2017).

### Statistical Analysis

The data analysis was conducted in SPSS version 21 (IBM Corp, 2012). The analyses carried out were (a) mixed-measures ANOVA, (b) repeated measures ANOVA, (c) multivariate analysis of variance (MANOVA), (d) one-way ANOVA. The aim of these analyses was to compare the performance between the three groups as well as the performance of each group in each condition of a test. Levene’s test was used to assess the equality of variances, Box’s Test for the assessment of the equivalence of covariance matrices, and Mauchly’s test of sphericity for the assessment of within-subject factor. Authors used Greenhouse-Geisser for thecorrection of sphericity violations. Partial eta-squared (η^2^) was used for the estimation of the effect size. Scheffe test (plus Bonferroni correction) was adopted for *post hoc* comparisons: even if there are issues related to test’s conservatism, given the exploratory ‘nature’ of this study, we considered Scheffe procedure as the best choice due to the following reasons: it tests all possible comparisons, it is robust in relation to non-normality, and it provides maximum protection against type I error, which was our main concern in this study (Armstrong and Hilton, 2006).

In the next step, data analysis was conducted in EQS version 6.1 (Bentler, 2005). Specifically, structural equation modeling (SEM) on covariance matrices was used. The ‘Robust maximum likelihood estimation’ procedure was performed due to small sample size and data kurtosis. The specific SEM technique applied to the data was path analysis. This technique was used to examine the directional relations between the constructs – observed variables which emerged from the measurements of each test (Kline, 2005). Lagrange Multiplier and Wald test (Brown, 2006) were conducted to investigate the elements of the model that didn’t fit the data well. In specific, a series of multiple-group path analysis were conducted to examine and compare the relations between the variables in the three groups, in order to reveal potential differences in the way participants of each grouprecruit executive functions when needed. Regarding the confirmation of a path model, a significance level of Goodness of Fit Index χ^2^ that is *p* > .05 is indicative of a good fit of the model to the data. In this study, authors calculated the Satorra-Bentler χ^2^ index (Bentler, 2005) due to the statistical analysis they chose to perform (Robust procedure). In addition, when the value of Root Mean Square Error of Approximation (RMSEA) is <0.05, it is also an indication of the good fit of the model to the data. RMSEA values ranging from 0.06 to 0.08 indicate a reasonable and therefore acceptable approximation error. RMSEA value is relatively “expanded” in cases of small sample size (*n* < 100) and that is reflected in confidence interval range (90% CI). This means that RMSEA should be considered as a model fit index, however, with caution (Bollen, 1990). Comparative Fit Index (CFI) examines whether the data fit a hypothesized measurement model compared to the basic model. Values greater than 0.90 indicate adequate fit of the model to the data, whereas values close to 1.00 indicate a good fit.

## Results

### Color – Word Interference Test, Standard Form: Inhibition and Switching

Initially, means and standard deviations (SD) of test scores in all C-WIT conditions were calculated for all groups. Subsequently, a series of analyses of variance (ANOVA) was applied in order to examine the ‘quantitative’ differences between the three groups in the variables under examination for the first three C-WIT conditions. Finally, a Multiple-Group Path Analysis was performed for the third C-WIT condition in order to examine the main differences between the three groups, in cases of the application of the executive function of inhibition.

#### Total Corrected and Non-corrected Errors

A 3 × 3 mixed design ANOVA (three groups of participants × three conditions of C-WIT) showed a significant main effect of the participant group, *F*(2,57) = 15.84, *p* < 0.0001, η^2^ = 0.35, condition, *F*(1.07,61.04) = 53.21, *p* < 0.0001, η^2^ = 0.48, as well as a significant group – condition interaction, *F*(2.14,61.04) = 21.21, *p* < 0.0001, η^2^ = 0.42.

With regards to the significant main effect of the participant group, Scheffe’s method of multiple comparisons showed that more statistically significant errors were made by the VaD group compared to the RVD group, *p* = 0.01, and to young adult group, *p* < 0.0001. The difference between the last two groups was non-significant, *p* = 0.057. A series of repeated measures ANOVA applied to the data in each group, showed that the group of young adults did not show statistically significant differences in the total number of errors in all three C-WIT conditions, *F*(1.23,23.49) = 2.13, *p* = 0.07. On the other hand, the group of adults with RVD showed differences in the errors in all three conditions, *F*(1.22,23.28) = 11.78, *p* = 0.001, with the highest number of errors (corrected and not corrected) being observed in the third condition, followed by the first and the second conditions. The VaD group showed significant differences in the total number of errors, *F*(1.00,19.17) = 39.55, *p* < 0.0001, with a greater number of errors being observed in the third C-WIT condition compared to the second condition (see **Table 2A**).

**Table 2A:** D-KEFS Color – Word Interference Test, Standard Form: comparisons of the three sample groups in total scores as well as of each group performance in the first three conditions.

Type of score		Group type		
		VaD^1^	RVD	YA
Total *n* of errors (*M* and *SD*)		2.71 (0.30)^∗^	1.36 (0.30)^∗^	0.31 (0.30)^∗^
Task completion time (*M* and *SD*)		73.46 (3.45)^∗^	60.26 (3.45)^∗^	33.75 (3.45)^∗^

**Group type**	**Type of score**	**Condition type**		
		**(1) Naming the colors**	**(2) Reading words in black**	**(3) Naming the dissonant ink colors (Inhibition)**

VaD	Total *n* of errors (*M* and *SD*)	0.10 (0.45)^∗^	0.00 (0.00)^∗^	8.05 (5.71)^∗^
	Task completion time (*M* and *SD*)	47.65 (2.28)^∗^	34.25 (1.58)^∗^	138.50 (9.47)^∗^
RVD	Total *n* of errors (*M* and *SD*)	1.05 (1.19)^∗^	0.10 (0.45)^∗^	2.95 (3.20)^∗^
	Task completion time (*M* and *SD*)	47.15 (3.18)^∗^	40.25 (4.66)^∗^	93.40 (7.95)^∗^
YA	Total *n* of errors (*M* and *SD*)	0.25 (0.55)	0.10 (0.31)	0.60 (0.88)
	Task completion time (*M* and *SD*)	30.35 (1.24)^∗^	22.45 (0.89)^∗^	48.45 (2.06)^∗^

*^1^VaD, older adults diagnosed with Vascular Dementia according to the criteria of probable Vascular Neurocognitive Disorder; RVD, community dwelling older adults having risk factors for vascular disease development; YA, young adults. ^∗^p < 0.017 (after Bonferroni correction).*

#### Task Completion Time

A 3 × 3 mixed design ANOVA was applied with the participant group (young adults, RVD, VaD) as a between-group variable and the C-WIT condition (first three conditions) as a within-group variable. The results of the analysis showed that there is a main group effect, *F*(2,57) = 34.25, *p* < 0.0001, η^2^ = 0.54, a main condition effect, *F*(1.01,62.94) = 212.84, *p* < 0.0001, η^2^ = 0.78, as well as a significant group – condition interaction, *F*(2.20,62.94) = 32.04, *p* < 0.0001, η^2^ = 0.52.

With regard to the main group effect, Scheffe’s multiple comparison showed that the VaD group had a significantly longer task completion time compared to young adults, *p* < 0.0001. The VaD group also differed from the young adult group, *p* < 0.0001.

For further analysis of the main condition effect, repeated measures ANOVA was applied to the data of each group. For the group of young adults, *F*(1.28,24.47) = 145.63, *p* < 0.0001, significant differences were observed in the completion time between the three conditions. Similar results were observed for both the RVD group, *F*(1.22,23.17) = 55.06, *p* < 0.0001, as well as for the VaD patients, *F*(1.03,19.70) = 109.28, *p* < 0.0001. However, despite the fact that the prototype of differences observed is almost similar among all groups, the young adult group spent much less time to complete the 3rd condition, compared to older adults with RVD and VaD (see **Table 2A**).

#### Multiple*-*Group Path Analysis Models for the 3rd C-WIT Condition (Inhibition)

**FIGURE 1:**
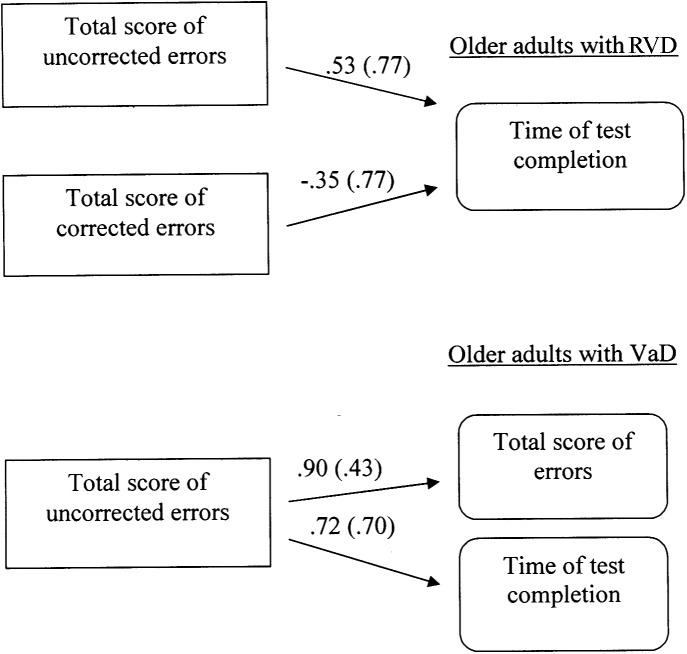
Multiple-group path model displaying differences in inhibitory control (3rd condition of Color – Word Interference Test) between older adults at risk of developing vascular disease (RVD) and Vascular Dementia (VaD) patients. All relationships are statistically significant for *p* < 0.05. Measurement error is given in parenthesis.

This analysis investigated the directed relations between the total number of errors (corrected and non-corrected), the total number of corrected errors, the total number of uncorrected errors and the completion time of the third condition in all three groups of participants. The results showed that different path models were confirmed for each group, Satorra-Bentler χ^2^ (3, *N* = 60) = 2.20, *p* = 0.53, CFI = 1.00, RMSEA = 0.00 (90% CI: 0.00 −0.19). In specific, regarding the group of young adults, no relations were observed between the measurements. However, the model for RVD adults appears to be interesting. In this case, it was found that the number of uncorrected errors is positively associated with the completion time (i.e., associated with increased time), while the number of corrected errors has a compensatory function, as associated with reduced completion time. Furthermore, a different model was confirmed for VaD adults, since the total number of uncorrected errors is positively associated with both the total number of errors and the completion time of the condition (see **Figure 1**).

#### Findings Regarding the Score in the 4th C-WIT Condition (Inhibition and Switching)

In this condition, only 8 participants from the group of VaD adults completed the test. Following the instructions, the fourth condition was not administered, because participants experienced great difficulty in the practice lines of this condition. For this reason it was not included in the aforementioned analyses. Regarding the group of RVD adults, although only one participant could not complete the fourth condition, the differences from young adults in both the number of errors and the completion time of the condition were statistically significant: Errors: *F*(1,38) = 21.71, *p* < 0.0001. Completion time: *F*(1,38) = 51.51, *p* < 0.0001 (see **Table 2B**).

**Table 2B:** D-KEFS Color – Word Interference Test, Standard Form: comparisons of the sample groups in the fourth condition: Switching between naming the color of the words and reading the words.

Type of score	Group type		
	VaD^1^	RVD	YA
Total *n* of errors (*M* and *SD*)	–	5.42 (4.11)^∗∗∗^	1.00 (1.00)^∗∗∗^
Task completion time (*M* and *SD*)	–	121.47 (40.27)^∗∗∗^	54.25 (11.33)^∗∗∗^

*^1^VaD, older adults diagnosed with Vascular Dementia according to the criteria of probable Vascular Neurocognitive Disorder; RVD, community dwelling older adults having risk factors for vascular disease development; YA, young adults. ^∗∗∗^p < 0.001.*

### Verbal Fluency Test: Inhibition and Switching

Means and SD were calculated for VFT conditions for all groups. Then, a series of analyses of variance was applied in order to examine the differences between the three groups on the relevant variables. Finally, based on Pearson correlations between variables and taking into account the sample size, a multiple*-*group path analysis was applied to the data of the second and third condition of VFT.

#### Total Correct Answers-Words

Initially, a 3 × 3 mixed design ANOVA was performed with the participant group (young adults, RVD, VaD) as a between-groups factor and the condition of VFT (three conditions) as a within-subjects factor. A significant main effect of the participant group was found, *F*(2,57) = 78.20, *p* < 0.0001, η^2^ = 0.73, as well as a main effect of the condition type, *F*(2,114) = 288.35, *p* < 0.0001, η^2^ = 0.83, and a significant group – condition interaction, *F*(2,114) = 11.95, *p* < 0.0001, η^2^ = 0.29.

Subsequently, analysis of variance was applied with the participant group as the independent variable and performance in each of the three conditions as the dependent variables: According to Pillai’s trace, the group effect was found to be significant, *V* = 0.78, *F*(2,57) = 12.03, *p* < 0.0001, η^2^ = 0.39. In particular, the group effect on the total number of correct responses in the phonemic fluency condition (1), *F*(2,57) = 42.95, *p* < 0.0001, η^2^ = 0.60, in the semantic fluency condition (2), *F*(2,57) = 56.13, *p* < 0.0001, η^2^ = 0.66, and in the semantic fluency condition under switching rules (3a), *F*(2,57) = 24.74, *p* < 0.0001, η^2^ = 0.46, was statistically significant. Scheffe’s *post hoc* comparison showed that young adults produced more words in the first condition, as compared to RVD and VaD groups, *p* < .0001. No significant differences were observed between the two older adult groups, *p* = 0.48. Similar results were observed in the second condition: young adults had a higher performance compared to RVD and VaD groups, *p* < 0.0001, whereas the difference between the two older adult groups was not significant, *p* = 0.13. It was found that in the condition ‘3a’ VaD adults produced a reduced number of words, compared to RVD, *p* = 0.016, and young adult groups, *p* < 0.0001. A significant difference was also observed between the last two groups, *p* < 0.0001 (see **Table 3**).

**Table 3:** D-KEFS: Verbal Fluency Test: Standard Form: comparisons of the three sample groups as well as of each group performance in the three conditions.

Condition type	Type of score	Group type		
		VaD^1^	RVD	YA
(1) Phonemic fluency	Total *n* of correct words (*M* and *SD*)	25.05 (4.33)^∗^	28.45 (10.76)^∗^	45.80 (6.18)^∗^
(2) Semantic fluency	Total *n* of correct words (*M* and *SD*)	22.95 (5.28)^∗^	27.10 (5.53)^∗^	43.00 (7.82)^∗^
(3a) Semantic fluency with switching	Total *n* of correct words (*M* and *SD*)	8.45 (0.80)^∗∗∗^	11.80 (0.70)^∗∗∗^	16.55 (0.93)^∗∗∗^
(3b) Semantic fluency with switching	Total *n* of switches (*M* and *SD*)	8.85 (0.83)^∗^	10.80 (0.83)^∗^	14.80 (0.83)^∗^

*^1^VaD, older adults diagnosed with Vascular Dementia according to the criteria of probable Vascular Neurocognitive Disorder; RVD, community dwelling older adults having risk factors for vascular disease development; YA = young adults. ^∗^p < 0.017; ^∗∗∗^p < 0.001.*

For a further analysis of the main condition effect repeated measures ANOVA was used to compare the data of each group. Young adults showed a statistically significant difference in the responses produced in all three conditions, *F*(2,38) = 138.90, *p* < 0.0001. In particular, they produced a greater number of correct words in the first and second condition, compared to the third, *p* < 0.0001. Similar results were observed for the RVD group, *F*(1.48,28.17) = 50.57, *p* < 0.0001, and also for the VaD group, *F*(2,38) = 150.52, *p* < 0.0001 (see **Table 3**).

#### Total Repetitions and Total Errors in the Three Conditions of VFT

A univariate analysis of variance showed that the three groups did not differ significantly in the total number of repetitions they did, *F*(2,57) = 1.86, *p* = 0.16, as well as in the total number of their errors, *F*(2,57) = 1.76, *p* = 0.18.

#### Total Number of Switches in the Third Condition (Semantic Fluency Under Rules of Switching) of VFT (Condition ‘3b’)

A univariate analysis of variance was performed, that showed a significant effect of the participant group on the total number of correct switches, *F*(2,57) = 13.09, *p* < 0.0001, η^2^ = 0.31. Scheffe’s multiple comparison showed that young adults achieved a significantly higher number of switches, compared to RVD and VaD groups, *p* = 0.006 and *p* < 0.0001, respectively (**Table 3**). However, there was no significant difference between the two groups of older adults, *p* = 0.27.

#### Multiple-Group Path Analysis Models for VFT

**FIGURE 2:**
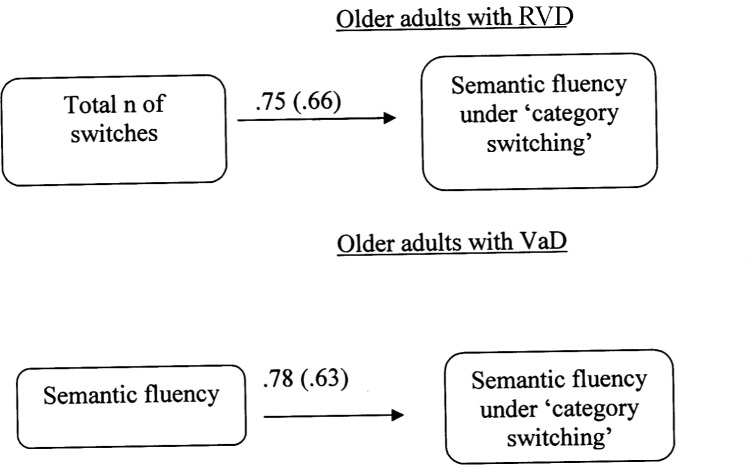
Multiple-group path model displaying differences in cognitive flexibility (Verbal Fluency Test: condition 3) between older adults at risk of developing vascular disease (RVD) and Vascular Dementia (VaD) patients. All relationships are statistically significant for *p* < 0.05. Measurement error is given in parenthesis.

A multiple-group path analysis was used to examine the directed relations between semantic fluency (2nd condition), the words produced in the condition of semantic fluency with switching (condition ‘3a’) and the total number of switches (condition ‘3b’). It must be noted that the error in measuring word production under switching condition was set to be equal in the two older adult groups. The data from young adults were not used in the analyses, because the aforementioned variable-scores were not significantly inter-correlated. The results for the two older adult groups, Satorra-Bentler χ^2^ (1, *N* = 40) = 0.70, *p* = 0.39, CFI = 1.00, RMSEA = 0.00 (90% CI: 0.00 -0.39), showed that for the RVD group it was confirmed a model in which the number of switches (condition ‘3b’) is positively associated with the production of words in the condition ‘3a’. On the other hand, a different model was confirmed for VaD adults. In this case, semantic fluency (2nd condition) appeared to be positively associated with the word production under switching rules (3a condition) (**Figure 2**).

##### Tower test: planning

Means and SDs were calculated for all measurements in all TT conditions for all groups. Subsequently, a series of analyses of variance was applied in order to examine the differences between the three groups of the sample on the variables under examination, namely the total number of problems given, the total number of violations, the precision of movements and the total achievement score. Based on the calculated Pearson correlations between variables of TT and taking into account the small sample size, a multiple-group path analysis was applied to the data of TT.

In more detail, analysis of variance was applied with the participants’ group as the independent variable and the four performance variables as dependent ones: the main effect of group was found to be significant, *V* = 0.85, *F*(2,57) = 10.33, *p* < 0.0001, η^2^ = 0.42. In particular, the group effect was significant on (a) the total number of problems given, *F*(2,57) = 17.47, *p* < 0.0001, η^2^ = 0.38, (b) the total number of rule violations, *F*(2,57) = 17.09, *p* < 0.0001, η^2^ = 0.37, (c) the precision of movements, *F*(2,57) = 11.79, *p* < 0.0001, η^2^ = 0.29, and (d) the total achievement score, *F*(2,57) = 33.45, *p* < 0.0001, η^2^ = 0.54.

Scheffe’s multiple comparison showed that VaD patients were given less problems (because of their inability to achieve the task or to resolve it correctly) compared to young adults and RVD group, *p* < 0.0001 and 0.001, respectively. Similar findings were found for the total number of violations, where significant differences were observed among all three groups, with the group of VaD patients showing the higher number of rule violations, *p* < 0.0001 and 0.004, respectively. A reduced precision of movements was observed in the VaD group compared to the other two groups, *p* < 0.0001 and 0.002, respectively. Finally, regarding the total achievement score, significant differences were observed among the three groups, with young adults to show higher score compared to RVD and VaD groups, *p* < 0.0001. The difference between the two older adult groups was also found to be significant, *p* = 0.008 (see **Table 4**).

**Table 4:** D-KEFS: Tower Test: comparisons of the three sample groups in four scores.

Type of score	Group type		
	VaD^1^	RVD	YA
Total *n* of problems (*M* and *SD*)	7.00 (0.23)^∗^	8.25 (0.23)^∗^	8.95 (0.23)^∗^
Total *n* of rule violations (*M* and *SD*)	8.85 (0.85)^∗^	4.80 (0.85)^∗^	1.80 (0.85)^∗^
Precision of movements (*M* and *SD*)	0.83 (0.15)^∗^	1.65 (0.15)^∗^	1.85 (.15)^∗^
Total achievement score (*M* and *SD*)	8.90 (0.74)^∗^	12.20 (0.74)^∗^	17.40 (.74)^∗^

*^1^VaD, older adults diagnosed with Vascular Dementia according to the criteria of probable Vascular Neurocognitive Disorder; RVD, community dwelling older adults having risk factors for vascular disease development; YA, young adults. ^∗^p < 0.013 (after Bonferroni correction).*

#### Multiple-Group Path Analysis Models for TT

**FIGURE 3:**
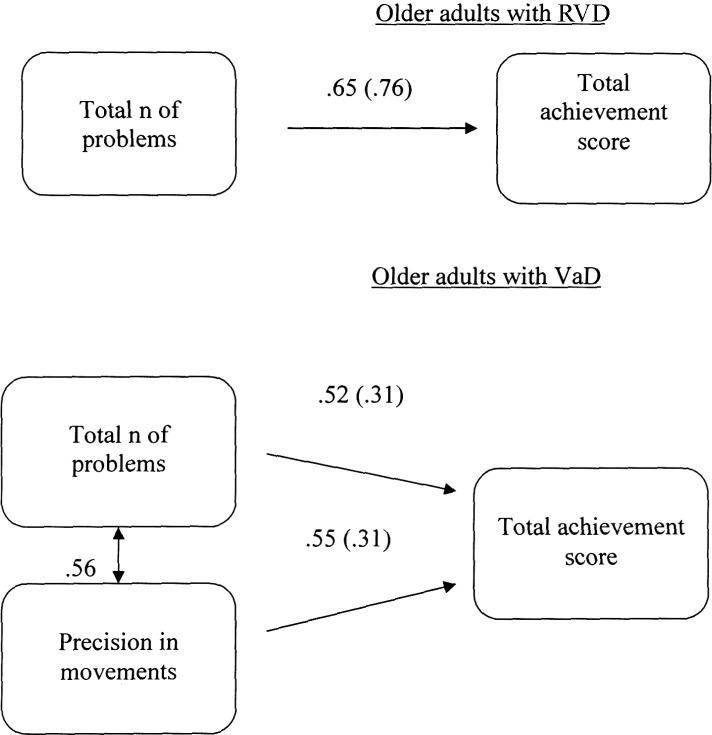
Multiple-groups path model displaying differences in cognitive planning (Tower Test) between older adults at risk of developing vascular disease (RVD) and Vascular Dementia (VaD) patients. All relationships are statistically significant for *p* < 0.05. Measurement error is given in parenthesis.

A multiple-group path analysis was applied to TT data to examine the directed relations between the total number of the problems given, the precision of movement and the total score achieved in all groups, for TT data. The results showed that different models were confirmed for each group, Satorra-Bentler χ^2^(1, *N* = 60) = 0.90, *p* = 0.34, CFI = 1.00 and RMSEA = 0.00 (90%CI: 0.00 −0.33). Regarding young adults, there was no relationship between measurements. For the RVD group, the confirmed model showed that only the total number of problems given was positively associated with the total score achieved. Regarding the VaD group, the confirmed model showed that both the total number of given problems and the precision of movements were positively linked to the total score achieved. There was also a significant correlation between the precision of movements and the number of problems given (see **Figure 3**).

## Discussion

### Cognitive Control in Vascular Dementia: Inhibition

Based on the extant literature supporting EF deficit in VaD, (Moorhouse et al., 2010; De Witte et al., 2011; Vasquez and Zakzanis, 2015; Moreira et al., 2017) the aim of this study was to go a step further in terms of examining whether older patients with incipient VaD would show a low performance in cold EF tasks mainly requiring the application of three types of executive functions, namely inhibition, switching (or set-shifting), and planning. The investigation of discrete EF abilities, in which older adults diagnosed with incipient VaD and community dwelling older adults with risk factors for vascular disease development (RVD) could show a pattern of ‘clear’ differences, was considered a subject with great interest, which would enable the development of a short and reliable diagnostic tool for early stage VaD.

*‘Inhibitory control’* is the process of altering one’s learned behavioral responses in a way that makes it easier to achieve a particular goal. In this vein, an inhibitory control task measures one’s ability to overcome their habitual or dominant response to a stimulus in order to implement a different response. In this study, inhibition was examined with the D-KEFS Color - Word Interference Test (Delis et al., 2001) which is based on selective information processing in the presence of disruptive stimuli.

According to the findings, the number, and mainly the type of errors (mostly uncorrected) in D-KEFS – C-WIT inhibition condition (3rd) might be an indicator of the differentiation between incipient VaD patients and community dwelling older adults presenting risk factors for vascular disease development. Theoretically, it has been argued that the ability to correct errors requires control and adjustment of behavior and is related to metacognitive skills which are impaired in old age (Delis et al., 2001). Thus the inability of VaD patients to correct their wrong responses may reflect a deficit in their metacognitive control system. Based on the findings, the first signs of such a deficit may be evident in community dwelling older adults with RVD, as compared to healthy young controls. However, it seems that at this phase individuals maintain their inhibitory control at a relatively adequate level, since they can correct a part of their errors, displaying metacognitive control to some extent, and perform well in tasks requiring inhibition.

With regards to the completion time, the findings indicated that in the inhibition condition, VaD patients took more time to complete the task, compared to young adults. According to Delis et al. (2001) persons who demonstrate a relatively good performance in naming and reading (1st and 2nd conditions), but have an increased response time and mainly make uncorrected errors in the inhibition condition, tend to be characterized by severe deficits in inhibitory control. Given the aforementioned findings, this pattern of performance is fully confirmed for VaD patients. In addition, first signs of deficit in inhibitory control, in terms of response time, /become visible in older adults with RVD when they are compared to young adults.

Taking into account the significantly increased overall response time and the total error score of VaD patients, in the D-KEFS – C-WIT compared to the other two groups, it can be suggested that *the Hypothesis 1 was partially confirmed* at least in the case of examining inhibitory control via a Stroop-type task: older adults diagnosed with incipient VaD have generally shown lower performance in the D-KEFS – C-WIT compared to older adults with RVD and young adults, while there are some indications of inhibitory control deficit in RVD group compared to young adults. In verification of these findings, Kramer et al. (2002) suggest that subcortical ischemic vascular disease is associated with impaired inhibition even in individuals without a diagnosis of dementia. It is noteworthy that in previous studies a significant contribution of the frontal and subcortical regions has been found in the ability to inhibit, (Lenartowicz et al., 2010) and especially of the anterior cingulate cortex, (Alvarez and Emory, 2006) and frontal gyrus (Leung et al., 2000).

A recent systematic review and meta-analysis study (Sudo et al., 2017) suggested a specific inhibitory control deficit in small vessel cognitive impairment (SVCI), based on the findings of a series of studies that used the Stroop test. The results of this review showed significant differences between SVCI patients and healthy controls in the number of errors as well as in the completion time of the 3rd condition of the Stroop test. The suggested interpretation for this deficit is that it is potentially due to vulnerability to interlobar disconnection on account of periventricular white matter abnormalities (Banich et al., 2000). Neurophysiological studies have indicated that periventricular white matter receives blood supply from terminal vessels of long perforating arteries in areas where branches of large cerebral vessels are encountered. However, most such arteries are very tortuous and this makes their areas vulnerable to hypoperfusion due to arteriosclerosis (Banich et al., 2000). In the same vein, another study (Dunet et al., 2016) which examined the relationship of cognitive impairment with functional connectivity between the basal ganglia and cingulate cortex in vascular parkinsonism, showed that inhibitory control and errors in the Stroop test are related to increased caudate nucleus functional connectivity with the perigenual anterior cingulate cortex, and decreased caudate nucleus functional connectivity with the posterior cingulate cortex at resting-state, respectively. White matter lesions, indicative of small vessel disease, were found to partially contribute in this pattern.

The D-KEFS VF was also used in this study to examine inhibitory control (see Materials and Methods). The findings showed that it is not able to differentiate VaD patients and older adults with RVD. A possible reason for this may be that D-KEFS VF doesn’t require the same level of inhibitory control as the D-KEFS C-WIT, while both older adult groups can function relatively well at the level required by D-KEFS VF. In any case, this finding is important, considering the widespread use of the verbal fluency task in neuropsychological examination as well as the findings of previous studies which showed a relationship of VaD and deficits in phonemic fluency (Duff Canning et al., 2004; Jones et al., 2006). However, the aforementioned meta-analysis and a series of studies on VCI did not replicate this relationship (Banich et al., 2000). Methodological reasons (e.g., different stage of VCI) may be responsible for this inconsistency.

### Cognitive Control in Vascular Dementia: Inhibition *plus* Switching

*‘Task or rule switching’* represents a kind of cognitive flexibility that involves the ability to shift attention between one task/rule and another. This ability allows a person to adapt efficiently to different environmental situations. In this study, switching was examined via the administration of the D-KEFS C-WIT (condition 4) and the D-KEFS VF (condition 3). Despite the fact that the two tests examine the same EF abilities, namely switching in conjunction with inhibitory control, it was considered that different results could be found due to the different lower-order cognitive abilities required to perform in each test.

In relation to the 4th condition of the D-KEFS C-WIT, the findings showed that community dwelling older adults with RVD presented a lower performance compared to young adults. The conclusion drawn is that the ability to control a situation declines when task requirements increase and this is particularly obvious in older adults. This finding seems to corroborate those of previous studies suggesting significant age-related effects on the combined application of inhibition and switching (Bryan and Luszcz, 2000; Plumet et al., 2005; Adornetti, 2016; Lam et al., 2017).

Nevertheless, in the present study, many VaD patients failed to complete the 4th condition. This is indicative of the increased difficulty of the patients with the specific neuropathology compared to community dwelling older adults, when they are asked to recruit two executive functions in combination, one of which had to be an expression of cognitive flexibility. Hence, it appears that failure to complete ‘inhibition plus switching’ condition of the D-KEFS C-WIT recommends a performance standard which can work as a ‘clear indicator’ of the differentiation between VaD patients and older adults with RVD.

However, with regard to the differentiation between the last group and young adults, confounding variables such as age-related factors and education should be taken into account, (Bryan and Luszcz, 2000; Plumet et al., 2005) in addition to potential early signs of neurodegeneration.

According to the findings, older adults with VaD and with RVD differentiated in the 3rd condition of the D-KEFS VF. Delis et al. (2001) claim that normal performance in phonological fluency (1st) and semantic fluency (2nd) conditions of the D-KEFS VF together with low performance in the semantic fluency under switching (3rd) condition indicate deficits in cognitive flexibility and not in verbal fluency. Based on the findings, it appears that such deficits are so severe in incipient VaD patients that they rely on their semantic knowledge to complete this condition without practically employing switching ability. Inversely, increased performance under switching recruitment (number of words generated) of older adults with RVD reflects that there is no such severe decline in set-shifting ability in this group, compared to VaD patients. On the other hand, the higher performance of young adults in the same condition compared to older adults with RVD, may indicate a progressive decline in cognitive flexibility along with age as well as its corresponding health deterioration (Bryan and Luszcz, 2000; Gunning-Dixon and Raz, 2003; Plumet et al., 2005; Spiro and Brady, 2008; Adornetti, 2016; Lam et al., 2017).

Several studies argue that there is an increase in interference and a decline in switching in people with severe white matter lesions (Lam et al., 2017). According to the findings of the same systematic review and meta-analysis study, (Sudo et al., 2017) which examined inhibitory control measured by the Stroop test, the decline of cognitive flexibility, as measured by the Trail Making Test – B in small vessel disease, also represents a specific EF deficit that might be attributed to the increased vulnerability to interlobar disconnection due to periventricular white matter hyperintensities.

Furthermore, Tsutsumimoto et al. (2015) used in their study the Trail Making Test to measure switching in MCI patients. According to their findings (gross) gray matter volume was both significantly and negatively associated with this EF ability. In the same light, another study in which gray matter correlates (regions of interest) of set-shifting in neurocognitive disorders was examined with the use of three D-KEFS tests [Design Fluency (non-verbal analog to Verbal Fluency), Trail Making, and Color-Word Interference] (Delis et al., 2001) and voxel-based morphometry, suggests that the switching performance correlated with focal regions in prefrontal and posterior parietal cortices. Interestingly, bilateral prefrontal cortex and the right posterior parietal lobe were identified as common sites for switching across all tasks. This finding is important as different types of tasks that measure set-shifting appear to have different underlying neuroanatomy to some extent (Kramer et al., 2007; Pa et al., 2010). Hence, VaD patients might experience lesions at least in the common areas that support switching, even from the initial stage of the disease.

In conclusion, the findings of this study showed that the level of switching ability can differentiate VaD even in the very first stage (Hypothesis 2). In fact, it appears that cognitive flexibility deficits are more severe and more obvious in incipient VaD compared to inhibitory control decline. Thus, the neuropsychological tests that are used to diagnose early stage VaD should include more than one conditions examining set-shifting.

### Cognitive Control in Vascular Dementia: Planning

*‘Cognitive planning’* encompasses the neuropsychological processes involved in the formulation, evaluation and selection of a sequence of thoughts and actions in order a desired goal to be achieved. It is considered a complex EF ability in terms of involving functions such as the ‘updating’ component of working memory, inhibitory control, and task/rule switching (Jefferson et al., 2006).

In this study, young adults and the two older adult groups showed a significant difference between them in two of the four examined variables (total achievement score and total rule violations). Community dwelling older adults with RVD were significantly worse than young adults in the aforementioned variables but they had almost the same level of precision in movements and solved the same number of Tower Test problems as young adults: this pattern of performance could be indicative of a progressive decline in planning along with age that starts with rule violations which indicate deficits in production and preservation (Miyake et al., 2000; Homack et al., 2005; Lee et al., 2009; Moreira et al., 2017). A step further, incipient VaD patients showed lower performance than the other two groups in all variables measured: this finding reflects a more generalized deficit of planning ability in VaD. Therefore, D-KEFS – TT appears to be a very promising tool for differentiating incipient VaD from vascular cognitive impairment in aging (Hypothesis 3).

Moreover, different patterns of variable associations were revealed in older adults with RVD and VaD patients: the more problems solved the higher the total score in the first older adult group. However, the performance of VaD patients was additionally affected by the precision in movements. According to Delis et al. (2001) a close-to-zero ratio in the precision in movements in combination with a low total achievement score indicate one’s inability to find correct problem-solving strategies as well as impairments in careful planning ability. VaD patients systematically presented this behavioral pattern. Furthermore, they failed to solve more complex problems, while the administration had to be interrupted whenever high-level planning was required.

The findings of the present study corroborate the results of previous research aimed at examining cognitive changes associated with moderate to severe white matter hyperintensities and less than 5 lacunes (Sudo et al., 2013, 2015). These studies indicated that a series of ‘impure’ EF tasks that assess complex EF abilities, such as planning, appear to be able to distinguish patients from healthy adults in the earliest pathological neuroimaging of cerebrovascular disease. Functional neuroimaging studies suggested that complex EF employ areas of an extensive neural network including prefrontal, parietal, medial and superior temporal cortices as well as subcortical structures. Leukoaraiosis as well as the onset of excessive white matter hyperintensities may lead to disconnection of those areas (Sudo et al., 2013, 2015).

With regards to Tower Test, in specific, fMRI studies indicated the involvement of the dorsolateral PFC (dlPFC) and anterior cingulate cortex (ACC) in the TT performance (Moreira et al., 2017). According to Foster et al. (2014) selective pyramidal cell atrophy in the dlPFC might partially explain the lower performance in TT. Aberrant functional connectivity between medial prefrontal cortex and ACC might also underlie the impaired complex EF ability (Zhou et al., 2016).

In conclusion, the present study shows that the D-KEFS – TT is an important tool for differentiating between incipient VaD and vascular aging of community dwelling older adults (Hypothesis 3).

Nevertheless, given that all EF tools used in this study are timed tests, processing speed could also be considered a function that is seriously affected in VaD. This is well-established in the extant literature and the main reason for this impairment is periventricular white matter hyperintensities (Gunning-Dixon and Raz, 2000; Oosterman et al., 2004; Vasquez and Zakzanis, 2015; Moreira et al., 2017; Sudo et al., 2017). Finally, it should be taken into account that the D-KEFS tests which were used in the present study measure cold EF mainly supported by dlPFC. However, it has been claimed that damage, at least in some central locations of the dlPFC, lead to defects in general intelligence (g) and not exclusively in EF (Barbey et al., 2013; Keifer and Tranel, 2013).

## Conclusion

At the theoretical level, the present study showed that deficits in complex or/and combined cold EF are more prominent in incipient VaD patients, compared to basic abilities of cognitive control. Specifically, the findings indicated that cognitive planning and cognitive flexibility are considerably affected by VaD progression even in the very first stages. On the contrary, inhibitory control does not seem to be a very useful indicator for the differential diagnosis of incipient VaD. At the applied level, this means that specific timed tests for the examination of planning and set-shifting should be ‘in a prominent position’ in the neuropsychological batteries administered for VaD evaluation. In this direction, the present study suggests some very specific indicators that can be used to differentiate incipient VaD from vascular aging experienced by community dwelling older adults (see **Table 5**), based on the findings which derived from a detailed examination of three EF abilities, that is planning, cognitive flexibility, and inhibitory control.

**Table 5:** Differences in inhibitory control, task/rule switching, and cognitive planning as indicators for differentiation between Vascular Dementia and vascular aging.

Vascular Dementia Vs. Vascular Aging
Color – Word Interference Test
• more errors
Color – Word Interference Test: inhibition (3rd condition)
• uncorrected errors → increased time to complete the condition vs. two types of errors → differential effects on response time
Color – Word Interference Test: inhibition *plus* switching (4th condition)
• failure to complete the condition vs. success to complete the condition

Verbal Fluency Test: inhibition *plus* switching (3rd condition)
• fewer words generated
• semantic fluency (2nd condition) → generating words (3a condition) *Vs.* n of correct switches (3b condition) → generating words (3a condition)

Tower test: planning
• greater number of violations
• fewer problems administered
• lower-level precision in movements
• lower total achievement score
• precision in movements and total n of problems administered → total achievement score *Vs.* total n of problems → total achievement score

However, this study can be only considered preliminary and replication is deemed necessary to ensure the reliability and validity of the findings.

### Limitations and Future Research

A number of limitations of the study have to be pointed out. First, the size of the sample was small due to the difficulty in recruiting participants diagnosed with incipient VaD as well as healthy controls of the same age, gender, and educational level. The administration of a broader battery of tools developed to measure specific EF abilities, not only cold but also hot EF, on a larger sample, may demonstrate more reliable and valid findings regarding executive functioning in vascular aging and incipient VaD. Moreover, it was also difficult to recruit a reliable number of patients with a diagnosis of a specific type of VaD. This is an important limitation, as different deficits have been reported on cognitive tests in specific types of VaD. As regards vascular aging, community dwelling older adult participants in the study reported that they have been diagnosed with specific risk factors. Hence, there was no objective examination and other risk factors for the development of vascular disease (e.g., diet, obesity) were not taken into account. The design of the study was cross-sectional and did not allow to ‘capture’ the trajectory of the change in vascular health during the lifespan. The cooperation of different scientific fields in a longitudinal study would enable a detailed observation of any changes occurring in the human brain, the way they are reflected on cognitive functions, and the time when the pathology starts. Other dementia groups should also be added to the sample, in order to differentiate VaD from other dementia types at the executive functioning level at an early stage of the disease. Moreover, more ecologically valid instruments or real life situations should be used to measure specific EF in the future.

## Author Contributions

KP designed the study under the supervision of DM, examined all participants, and participated in the statistical processing of the data and the writing of the manuscript. OS and VP participated in the writing of the manuscript. DS corrected the writing style of the manuscript. VC provided all patients diagnosed with VaD by her Clinic as well as all participants with vascular risk factors. GP participated in the statistical processing of the data and provided the main neuropsychological instrument of the study. DM brought the general supervision in all phases of the study which is a part of a broader research project of the same as principal investigator.

## Conflict of Interest Statement

The authors declare that the research was conducted in the absence of any commercial or financial relationships that could be construed as a potential conflict of interest.
